# Genome-wide identification and characterization of *14-3-3* gene family related to negative regulation of starch accumulation in storage root of *Manihot esculenta*

**DOI:** 10.3389/fpls.2023.1184903

**Published:** 2023-08-29

**Authors:** Ranran Pan, Yajie Wang, Feifei An, Yuan Yao, Jingjing Xue, Wenli Zhu, Xiuqin Luo, Hanggui Lai, Songbi Chen

**Affiliations:** ^1^ Tropical Crops Genetic Resources Institute, Chinese Academy of Tropical Agricultural Sciences/Key Laboratory of Ministry of Agriculture for Germplasm Resources Conservation and Utilization of Cassava, Haikou, China; ^2^ College of Tropical Crops, Hainan University, Haikou, China; ^3^ Institute of Tropical Bioscience and Biotechnology, Chinese Academy of Tropical Agricultural Sciences/Key Laboratory of Ministry of Agriculture of Biology and Genetic Resources of Tropical Crops, Haikou, China

**Keywords:** cassava, *Me14-3-3* genes, gene expression, overexpression, VIGS, starch accumulation

## Abstract

The 14-3-3 protein family is a highly conservative member of the acid protein family and plays an important role in regulating a series of important biological activities and various signal transduction pathways. The role of 14-3-3 proteins in regulating starch accumulation still remains largely unknown. To investigate the properties of 14-3-3 proteins, the structures and functions involved in starch accumulation in storage roots were analyzed, and consequently, 16 *Me14-3-3* genes were identified. Phylogenetic analysis revealed that Me14-3-3 family proteins are split into two groups (ε and non-ε). All Me14-3-3 proteins contain nine antiparallel α-helices. Me14-3-3s-GFP fusion protein was targeted exclusively to the nuclei and cytoplasm. In the early stage of starch accumulation in the storage root, *Me14-3-3* genes were highly expressed in high-starch cultivars, while in the late stage of starch accumulation, *Me14-3-3* genes were highly expressed in low-starch cultivars. *Me14-3-3 I*, *II*, *V*, and *XVI* had relatively high expression levels in the storage roots. The transgenic evidence from *Me14-3-3II* overexpression in *Arabidopsis thaliana* and the virus-induced gene silencing (VIGS) in cassava leaves and storage roots suggest that *Me14-3-3II* is involved in the negative regulation of starch accumulation. This study provides a new insight to understand the molecular mechanisms of starch accumulation linked with *Me14-3-3* genes during cassava storage root development.

## Introduction

Cassava accumulates much starch in its storage root; it is one of the most important root crops in the world; and it is a staple food crop in tropical Africa (De Souza et al., 2017; Jia et al., 2011; An et al., 2019). The formation and development of storage roots are important factors that affect cassava yield. Tuberization of cassava roots can be divided into three stages, namely, the tuber initial stage, expanding stage, and maturity stage (Yao et al., 2014). Cassava microarray data showed that the glycolysis/gluconeogenesis pathway, starch and sucrose metabolism pathway, and pentose phosphate pathway might be involved in the development of storage roots. Comparative proteomics found that a total of 154 differentially expressed proteins were identified during cassava root tuberization (Sojikul et al., 2010) and were involved in starch and sucrose metabolisms. One of the Me14-3-3 proteins had a significant change in the level of expression and phosphorylation during the tuber expanding stage, and the gene encoding this protein was transferred to *Arabidopsis*, and the regulating function of starch accumulation was also verified (Wang et al., 2016).

14-3-3 proteins are specialized adapter proteins that regulate the activities of the phosphorylated target protein by protein–protein interactions (Yang et al., 2019; Zhao et al., 2021). In plants, 14-3-3 proteins are encoded by a gene family. The abundance of the 14-3-3 protein family in plants is different, and there are various expression patterns of different members in plant tissues. Plant 14-3-3 proteins can be divided into the ε and non- ε subgroups (Xu et al., 2016). A previous report showed that at least eight subtypes of 14-3-3 proteins were identified in rice, and their roles and expression patterns are very different in different tissues; for example, OsGF14b is abundant in roots, low in stems and leaves, and almost absent in seeds and glumes. OsGF14d and OsGF14g show similar expression patterns, with the highest expression in mature leaves and stems and low expression in seeds and glumes (Yao et al., 2007; Yashvardhini et al., 2017; Zhang et al., 2019). Recent studies demonstrated that 14-3-3 proteins in plants are involved in regulating plant development, stress responses, and metabolism (Ormancey et al., 2017). The biological functions of the plant 14-3-3 proteins are related to regulating the phosphorylation of the target proteins (Denison et al., 2011; Boer et al., 2013; Yang et al., 2019). In *Arabidopsis*, the x/ψ/κ 14-3-3 can interact with AGT3 and S-adenosine methionine synthetase that are involved in plant nutrition absorption, affecting plant life development (Ryoung et al., 2011). Some 14-3-3 proteins in plants play a very important role in response to environmental stresses, such as in tomatoes (Xu and Shi, 2006) and cotton (Sun et al., 2011). It has been reported that 14-3-3 proteins are also involved in the carbohydrate metabolism of crops, such as potatoes (Magdalena et al., 2005) and barley (Morris, 2010). In rice, a member of 14-3-3 protein GF14f interacts with enzymes in sucrose breakdown, starch synthesis, tricarboxylic acid (TCA) cycle, and glycolysis to negatively affect grain development and filling (Zhang et al., 2019).

However, the structures and subtypes of 14-3-3 proteins (Me14-3-3) in cassava are not well known. The expression levels of Me14-3-3 proteins in each development stage of cassava storage roots and their mechanisms in the processes of starch accumulation in storage roots are still unclear. In the present study, the structures and subtypes of Me14-3-3 proteins were analyzed, and the expression patterns and the subcellular locations of Me14-3-3 proteins in each stage of cassava storage root development were investigated. These results will provide a new clue to understanding the molecular mechanisms of carbohydrates and starch accumulation during cassava storage root development.

## Materials and methods

### Plant materials and growth conditions

*Nicotiana benthamiana* plants were grown in soil in a growth chamber at 26°C, 80% humidity, under a 16-h light/8-h dark cycle. The seedlings of *N. benthamiana* used for transient expression were grown for 6–7 weeks, which grew robustly and disease-free.

The two cassava cultivars SC6068 and SC5 were selected in the present study. Their starch content data were obtained from Ye (2017), SC6068 < SC5. The stem cuttings of SC6068 and SC5 were planted in the fields of the National Cassava Germplasm Repository (NCGR), Danzhou, China. The storage roots were classified into three stages, namely, the root initial stage (RIS; planting for 120 days), root expanding stage (RES; planting for 210 days), and root maturity stage (RMS; planting for 300 days). The storage roots of SC6068 and SC5 in the stages of RIS, RES, and RMS were collected for RNA extraction to analyze the differential expressions of *Me 14-3-3* genes. The experiment was carried out in the Ministry of Agriculture and Rural Affairs Key Laboratory of Protection and Utilization of Cassava Germplasm Resources from April 2018 to April 2019. Each stage of the storage root of SC6068 and SC5 was used as one treatment, and each treatment included 10 plants. Three biological replicates were performed for each treatment. All samples were immediately frozen in liquid nitrogen upon collection and stored at −80°C.

### Identification and sequence analysis of *Me14-3-3* genes in cassava

Cassava 14-3-3 proteins were identified in the phytozome v10 database (using 14-3-3 as the keyword search on the website https://phytozome.jgi.doe.gov/pz/portal.html#!info?alias=Org_Mesculenta) (Goodstein et al., 2012). *Me14-3-3* genes were amplified based on the search sequences to design primers by using Primer 5.0. These primer sequences are listed in **Supplementary Table S4**. The PCRs were carried out in a final volume of 50 µL, containing 1 µL of cDNA from different tissues, by following the manufacturer’s instructions of the Ex Taq DNA polymerase kit (Takara, Maebashi, Japan). The PCR cycling conditions were as follows: 3 min at 94°C, followed by 30 cycles of 94°C for 30 s, a range of annealing temperatures for different types of *Me14-3-3s* from 57°C to 63°C for 30 s, 72°C for 1 min, and a final extension of 10 min at 72°C. The PCR products were separated on 1% agarose gel and purified by an Axygen Purification kit (Axygen, Union City, CA, USA), cloned into pMD19-T vector (Takara, Japan), and sequenced (The Beijing Genomics Institute, Guangzhou, China). All of *Me 14-3-3* genes were analyzed by using ORF finder (https://www.ncbi.nlm.nih.gov/orffinder/) software and the physicochemical properties of amino acids were analyzed by using the ProtParam tool (http://www.expasy.ch/tools/protparam.html) software.

### Gene structure and phylogenetic tree analysis

Sequences of *Me14-3-3* genes were obtained from the cassava database. The exon/intron organization of *Me14-3-3* genes was identified by using the GSDS website (http://gsds2.cbi.pku.edu.cn) (Guo et al., 2007). The phylogenetic tree of the Me14-3-3 protein family was constructed by MEGA7.1 software using the neighbor-joining method with 1,000 bootstrap replicates (Tamura et al., 2011). Genes with 99% bootstrap values were determined to be sister–gene pairs. The known 14-3-3 protein sequences from soybean, *Arabidopsis*, and rice and other nine plants obtained from the National Center for Biotechnology Information (NCBI) database were used as query sequences when conducting a BLAST search against the public genomic database (http://phytozome.jgi.doe.gov/pz/portal.html). The accession numbers of 14-3-3 proteins from *Arabidopsis*, soybean, rice, and other eight plants are listed in **Supplementary Table S3**.

### Cassava *14-3-3* gene family subcellular localization

Specific primers were designed with the Primer 5.0 software (**Supplementary Table S4**), and the specific target fragments of *ME14-3-3* genes were amplified with the high-fidelity enzyme Prime STAR^®^ Max DNA Polymerase and then inserted into the plasmid pCAMBIA1300-35S-GFP to pCAMBIA1300-35S-Me14-3-3-GFP as positive plasmids. The control plasmid pCAMBIA1300-35S-GFP and the positive plasmid pCAMBIA1300-35S-Me14-3-3-GFP were transferred into an *Agrobacterium tumefaciens* strain LBA4404 by the freeze–thaw method, respectively. The leaves of tobacco were infected with the transformed *A. tumefaciens* LBA4404 using the method described in Wu et al. (2010) and then photographed under a laser confocal microscope after 3 days. The excitation wavelength and emission length of blue fluorescent protein were 514 and 527 nm, respectively. The excitation wavelength of green fluorescent protein was 490 nm, and the emission length was 509 nm.

### RNA isolation and qRT-PCR analysis

Total RNAs were extracted from frozen storage roots in the stages of RIS, RES, and RMS of cassava cultivars SC6068 (low starch content) and SC5 (high starch content) using Rneasy plant mini kits (Qiagen, Hilden, Germany). cDNAs were synthesized using a PrimeScript cDNA Synthesis kit (Takara, Japan). Quantitative RT-PCRs were performed on a CFX96 (Bio-Rad, Hercules, CA, USA) using the SYBR Green Supermix, and the primers are listed in **Supplementary Table S4**. The PCR cycling conditions were 95°C for 3 min and then followed by 40 cycles of 95°C for 10 s and 60°C for 30 s. After each PCR run, a dissociation curve of transgenic lines was generated to confirm the specificity of the product. Three biological replicates with three technical replicates were performed for each reaction. The relative expression levels of the target genes were assessed based on the 2^−ΔΔ^ *^C^* ^t^ method.

### A cassava *14-3-3* gene overexpression in *Arabidopsis thaliana*

The coding region of *14-3-3II* was isolated from cassava with primers listed in **Supplementary Table S4**. The *14-3-3II* gene was subcloned into the P-super 1300+ vector between the *Xba*I and *Kpn*I sites, resulting in a construct for overexpression of *14-3-3II* gene under the control of the CaMV 35S promoter in plants. The construct was introduced into the *A. tumefaciens* strain GV3101 through electroporation. The *A. thaliana* ecotype Columbia (Col) was transformed *via* the floral dip method (Li et al., 2014). The *Arabidopsis* inflorescence was completely immersed in the infection solution and then removed after infection for 1 min. The infected *Arabidopsis* plants were placed in dark and moist boxes and removed for 24 h for continued light culture. Infected once every 2 days, after about five times, the newly growing inflorescence was removed. Once the fruit pods were full, the seeds were treated by drought, and then the seeds were collected. T3 homozygous *Arabidopsis* transgenic lines (1#, 3#, and 4#) were used for further experiments.

### Virus-induced gene silencing of *Me14-3-3II* in cassava

Cassava SC5 and SC6068 were planted in the soil containing vermiculite and nutrient soil in equal ratios and grown in a greenhouse at 26°C–28°C and a 12-h light/12-h dark cycle. After 40 days, they were used for virus-induced gene silencing (VIGS). For vector construction, 300-bp *Me14-3-3II* DNA fragments were cloned into pCsCMV-NC as described by Tuo et al. (2021). The primers (14-3-3-vigs-F: agtggtctctgtccagtcctCAGGATATTGCAAATGCAGA; 14-3-3-vigs-R: ggtctcagcagaccacaagtTCACTGCTGTTCATCGGTGG) were provided by Tsingke Biotechnology Co., Ltd. (Beijing, China). The *A. tumefaciens* strain GV3101 was co-transformed using the recombinant plasmids and further cultured at 28°C before resuspension in 10 mM of MgCl_2_, 10 mM of MES, and 20 mM of acetosyringone. These preparations were injected into the cassava plants at the back of the leaves using a 1-mL syringe and allowed to grow in the greenhouse before further analyses (Yan et al., 2021). Twenty-one days after injection, the leaves of cassava were collected to detect the effects of VIGS on *Me14-3-3II* expression by qPCR. The same leaves were also used to analyze the contents of starch, sucrose, and glucose. Cassava SC5 and SC6068 *Me14-3-3II*-silenced seedlings were cultivated in the field located in the National Cassava Germplasm Bank, CATAS, and harvested after 4.5 months in the field.

### Statistical analysis

SPSS 16.0 was used for statistical analysis. All data were expressed as the mean ± standard error (SE). All data were from three biological replications. The values with different letters are significant differences (p < 0.05) according to a one-way ANOVA.

## Results

### Identification and characterization of *Me14-3-3* genes in cassava

A total of 16 genes from the *Me14-3-3* family collected from the phytozome v10 database using the Pfam ID of 14-3-3 protein (PF00244) as keywords were obtained and cloned, and named *Me14-3-3I* to *XVI*. The *Me14-3-3* gene reported by Wang et al. (2016) was named *Me14-3-3 VII*. Full-length coding sequences of the *Me14-3-3* genes range from 396 bp (*Me14-3-3II*) to 795 bp (*Me14-3-3VII*, *Me14-3-3VIII*, and *Me14-3-3XI*). The sizes of the deduced Me14-3-3 proteins varied between 131 and 264 amino acids (aa), with an average of 247 aa. Their molecular weights (Mws) varied from 14.71 to 30.05 kDa, and the theoretical pI ranged from 4.57 to 4.98 (**Table 1**). Among the Me14-3-3 proteins, 12 members had an instability index greater than 40, and four members had an instability index less than 40, which indicates that most of the members are unstable proteins.

**Table 1:** Characterization of the *Me14-3-3* genes identified in the present study.

Gene name	Locus ID	ORF length (bp)	Amino acid length (aa)	Mw (kDa)	PI	Instability index (Gamage et al., 2019)
*Me14-3-3I*	Manes.18G060900	762	253	28.61	4.78	41.32
*Me14-3-3II*	Manes.18G065000	396	131	14.71	4.57	34.88
*Me14-3-3III*	Manes.12G127800	759	252	28.5	4.75	42.27
*Me14-3-3IV*	Manes.01G028600	711	236	26.82	4.88	47.00
*Me14-3-3V*	Manes.02G146600	762	253	28.49	4.72	39.24
*Me14-3-3VI*	Manes.18G066900	699	232	26.24	4.98	46.63
*Me14-3-3VII*	Manes.01G108700	795	264	29.83	4.75	47.84
*Me14-3-3VIII*	Manes.02G067200	795	264	29.8	4.79	50.39
*Me14-3-3IX*	Manes.01G133100	777	258	29.27	4.84	36.44
*Me14-3-3X*	Manes.02G151900	762	253	28.97	4.77	53.53
*Me14-3-3XI*	Manes.05G108900	795	264	30.05	4.89	47.32
*Me14-3-3XII*	Manes.10G104500	783	260	29.35	4.77	47.80
*Me14-3-3XIII*	Manes.07G035100	786	261	29.45	4.76	45.32
*Me14-3-3XIV*	Manes.15G093600	783	260	29.61	4.85	46.03
*Me14-3-3XV*	Manes.02G149900	780	259	29.31	4.72	47.79
*Me14-3-3XVI*	Manes.02G091000	777	258	29.22	4.72	37.91

**Figure 1:**
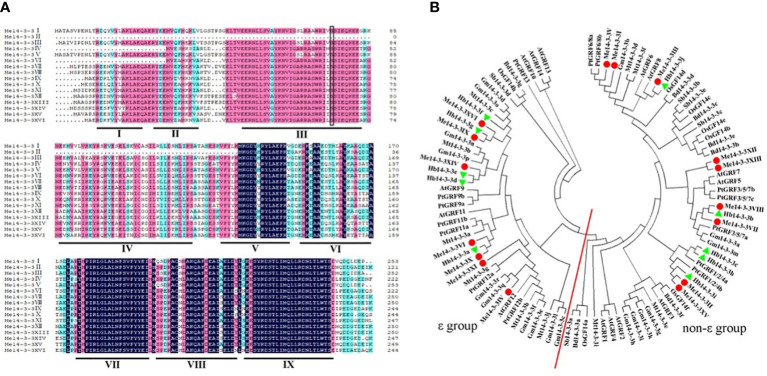
Alignment of the Me14-3-3 proteins and phylogenetic tree of 14-3-3 homologs. **(A)** Alignment of the Me14-3-3 proteins using the DNAMAN program. Dark-blue shading, pink shading, and light blue shading represent 100%, 75%, and 50% amino acid residue similarities, respectively. The I–IX domains of Me14-3-3 proteins are labeled to be the α-helices. The black box indicates the conserved phosphorylation sites in the Me14-3-3 proteins. **(B)** Phylogenetic trees of the 14-3-3 family proteins in nine plants constructed using the MEGA7.1 program. The red line separates the ϵ and non-ϵ groups.

Alignment of all members of the Me14-3-3 proteins shows low similarity at the N-terminal amino acid sequence and higher identity at the C-terminus (**Figure 1A**). The entire Me14-3-3 family proteins showed 71.97% similarity. The C-terminus plays an important role in calcium binding and dimer formation (Brian et al., 2006). 14-3-3 proteins contain nine antiparallel α-helices (Gardino et al., 2006). Similarly, most Me14-3-3 proteins are composed of nine α-helices, while Me14-3-3II loses the α-helices of Me14-3-3I, II, III, IV, and part of V. Me14-3-3IV and Me14-3-3 VI lose the α-helices of Me14-3-3I, and part of II. The α-helices of 14-3-3I–IV are thought to be involved in dimerization (Aitken, 2006). Therefore, it can be speculated that the protein dimerization ability of Me14-3-3II, IV, and VI may be weaker. The α-helices of Me14-3-3I, II, III, IV, and VI show a little bit of similarity, while the α-helices of Me14-3-3V, VII, VIII, and IX have an extensive identity. This phenomenon was also found in other 14-3-3 proteins, such as 14-3-3 proteins from *A. thaliana* (Chung et al., 1999). The divergent termini may relate to functional divergence, which could give each protein its specific function by binding a range of possible target proteins.

### Phylogenetic relationship of Me14-3-3 proteins

To investigate the phylogenetic relationships of Me14-3-3 proteins, a phylogenetic tree was constructed based on the full-length sequence alignments of 100 members of 14-3-3 proteins from cassava, *Arabidopsis*, rice, and other nine species. The gene names and locus IDs of these 14-3-3 proteins are listed in **Supplementary Table S1**. According to phylogenetic analysis, these 14-3-3 proteins are divided into two subfamilies (**Figure 1B**). According to previous reports, subfamilies are designated as the ϵ and non-ϵ groups (Xu et al., 2016). Seven Me14-3-3 proteins of Me14-3-3XVI, IX, XIV, VI, X, XI, and IV are located in the ϵ group in which Me14-3-3XVI and IX, Me14-3-3X, and VI have a close relationship. Nine Me14-3-3 proteins, namely, Me14-3-3V, I, III, XII, XIII, VIII, VII, II, and XV are located in the non-ϵ group, which are clustered into four subclasses. Most Me14-3-3 proteins have a close relationship with 14-3-3 proteins from *Hevea brasiliensis*, in which the two species belong to Euphorbiaceae.

### Gene structures of *Me14-3-3* genes

**Figure 2:**
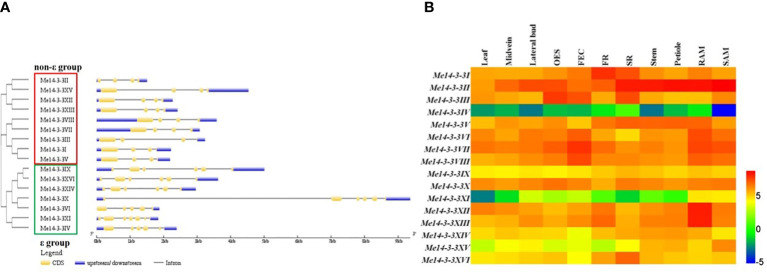
Exon–intron structures and expression analyses of the *Me14-3-3* genes. **(A)** Exon–intron structures of the *Me14-3-3* genes according to their phylogenetic relationships. An unrooted phylogenetic tree was constructed with 1,000 bootstraps based on the full-length sequences of *Me14-3-3* genes. Exon–intron structure analyses of the *Me14-3-3* genes were performed using the online tool GSDS. The lengths of the exons and introns of each *Me14-3-3* gene are drawn to scale. **(B)** Expression analyses of the *Me14-3-3* genes in different tissues of cassava cultivar TME 204. The heat map was created according to the log2-fragments per kilobase of transcript per million fragments mapped (FPKM) value of *Me14-3-3*s. Changes in gene expression are shown in color as the scale. OES, organized embryogenic structure; FEC, friable embryogenic callus; FR, fibrous root; SR, storage root; RAM, root apical meristem; SAM, shoot apical meristem.

To further explore the relationships among the different types of *Me14-3-3s*, the gene structures (exon/intron organization) were analyzed according to the alignment analysis of the cloned sequences with genomic sequences from the cassava genome database. The results showed that members of the Me14-3-3 family were split into the ϵ and non-ϵ groups (**Figure 2A**). Most of the ϵ group genes are encoded by six exons, except for *Me14-3-3VI* (five exons) and *Me14-3-3XI* (seven exons), while all the non-ϵ group genes are encoded by four exons. Generally, the *Me14-3-3* genes within a subclass have similar gene structures. Interestingly, *Me14-3-3VI* and *Me14-3-3X*, located in the same subclass, have different gene structures, for which *Me14-3-3X* is encoded by six exons, and its first intron is the longest intron among all the *Me14-3-3s*, while *Me14-3-3VI* is encoded by five exons.

### Expression analyses of *Me14-3-3* genes in various tissues of cassava plant

To study the expression of *Me14-3-3* genes in different tissues, RNA-seq data from 11 tissues of cassava cultivar TME204 were analyzed (Wilson et al., 2017). These tissues include leaves, midveins, lateral buds, organized embryogenic structures, friable embryogenic calli (FECs), fibrous roots (FRs), storage roots (SRs), root apical meristems (RAMs), and shoot apical meristems (SAMs). Fragments per kilobase of transcript per million mapped reads (FPKM) values of the *Me14-3-3* genes were used to generate a heat map (**Figure 2B**, **Supplementary Table S2**). Four genes (*Me14-3-3IV*, *Me14-3-3IX*, *Me14-3-3XI*, and *Me14-3-3XV*) have low expression levels across all the analyzed tissues, especially in which *Me14-3-3IV* and *Me14-3-3XI* are barely expressed. *Me14-3-3II* has high expression in most tissues. Some gene expressions are tissue specific. *Me14-3-3XII* and *Me14-3-3XIII* have relatively high expression levels in fibrous roots and root apical meristems in comparison with other tissues, in which these two genes may be involved in root development and nutrient uptake. *Me14-3-3VI*, *Me14-3-3VII*, and *Me14-3-3VIII* have relatively high expression levels in organized embryogenic structures (OESs), FECs, RAMs, and SAMs in comparison with other tissues, in which these genes may be related to cell division activity. *Me14-3-3I*, *II*, *V*, and *XVI* have relatively high expression levels in storage roots, in which these four genes may be involved in starch accumulation and storage root formation.

### Expression analysis of *Me14-3-3* genes in storage root of different cassava cultivars

**Figure 3:**
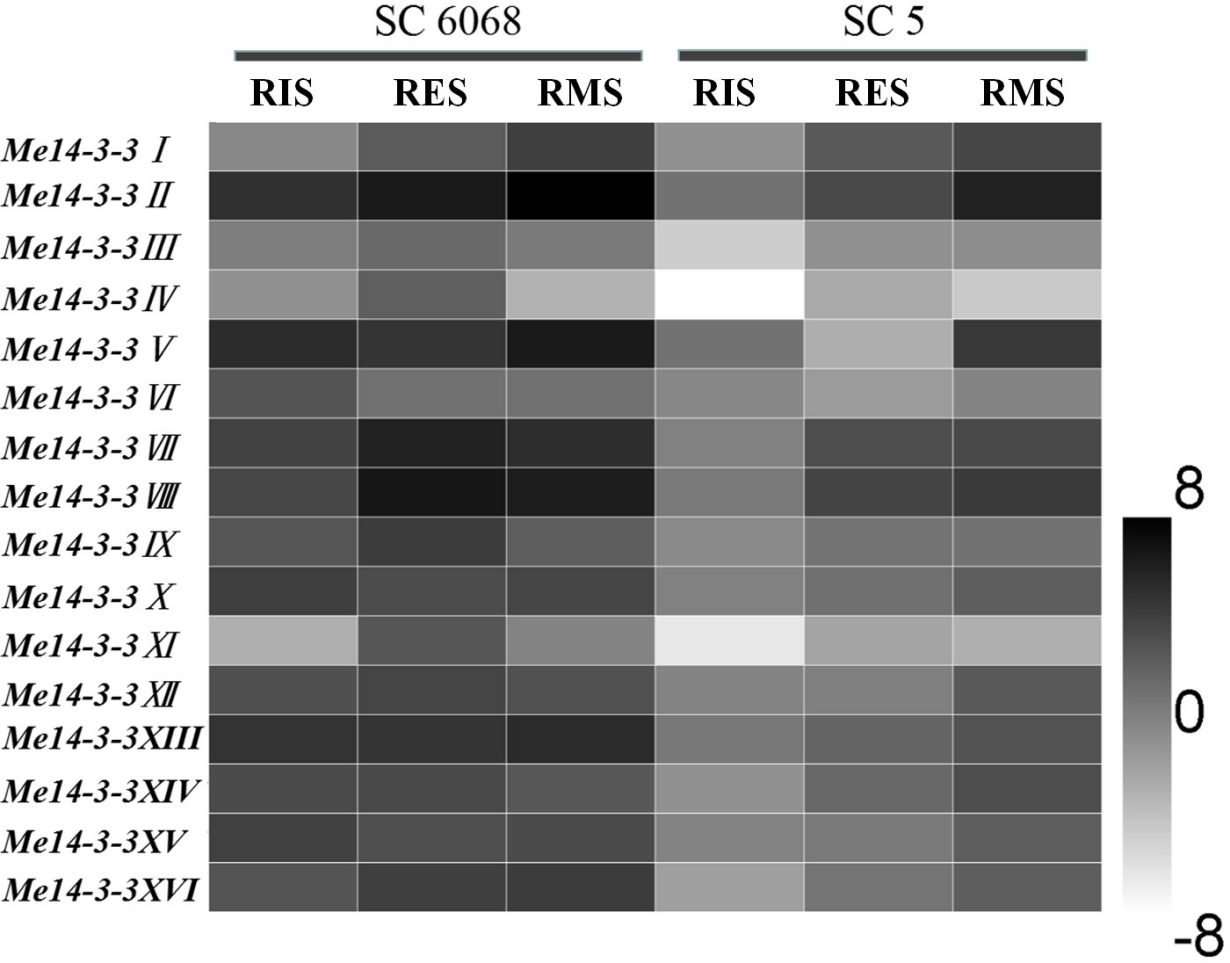
Expression analysis of *Me14-3-3s* during starch accumulation in cassava storage roots. The mean fold changes of each gene between different stages of storage root development in the cassava cultivars of SC6068 and SC5 were used to calculate its log2-relative expression levels. Data are the mean ± SD of n = 3 biological experiments. RIS, root initial stage; RES, root expanding stage; RMS, root maturity stage.

To investigate the role of *14-3-3* genes during root development, their expression patterns in two cassava cultivars, namely, SC6068 (low starch content) and SC5 (high starch content), were determined using qRT-PCR. Storage roots in RIS, RES, and RMS were collected for testing. The results showed that the expressions of *Me14-3-3* genes in the SC6068 cultivar are higher than those in SC5 during all development stages (**Figure 3**). In the SC5 cultivar, the expression levels of *Me14-3-3* genes are the lowest at the RIS stage and gradually increased as the storage roots are expanding and accumulating more starch at RES and RMS stages. However, most *Me14-3-3* genes in the SC6068 cultivar maintain high expression levels at RIS, RES, and RMS stages. Five genes, *Me14-3-3II*, *V*, *VII*, *VIII*, and *XIII*, showed higher expression levels than the rest of the *Me14-3-3* members in all tested samples. According to these results, it can be speculated that some of the Me14-3-3 proteins may play an important role in cassava root tuberization and starch synthesis.

### Subcellular localization of Me14-3-3 proteins

**Figure 4:**
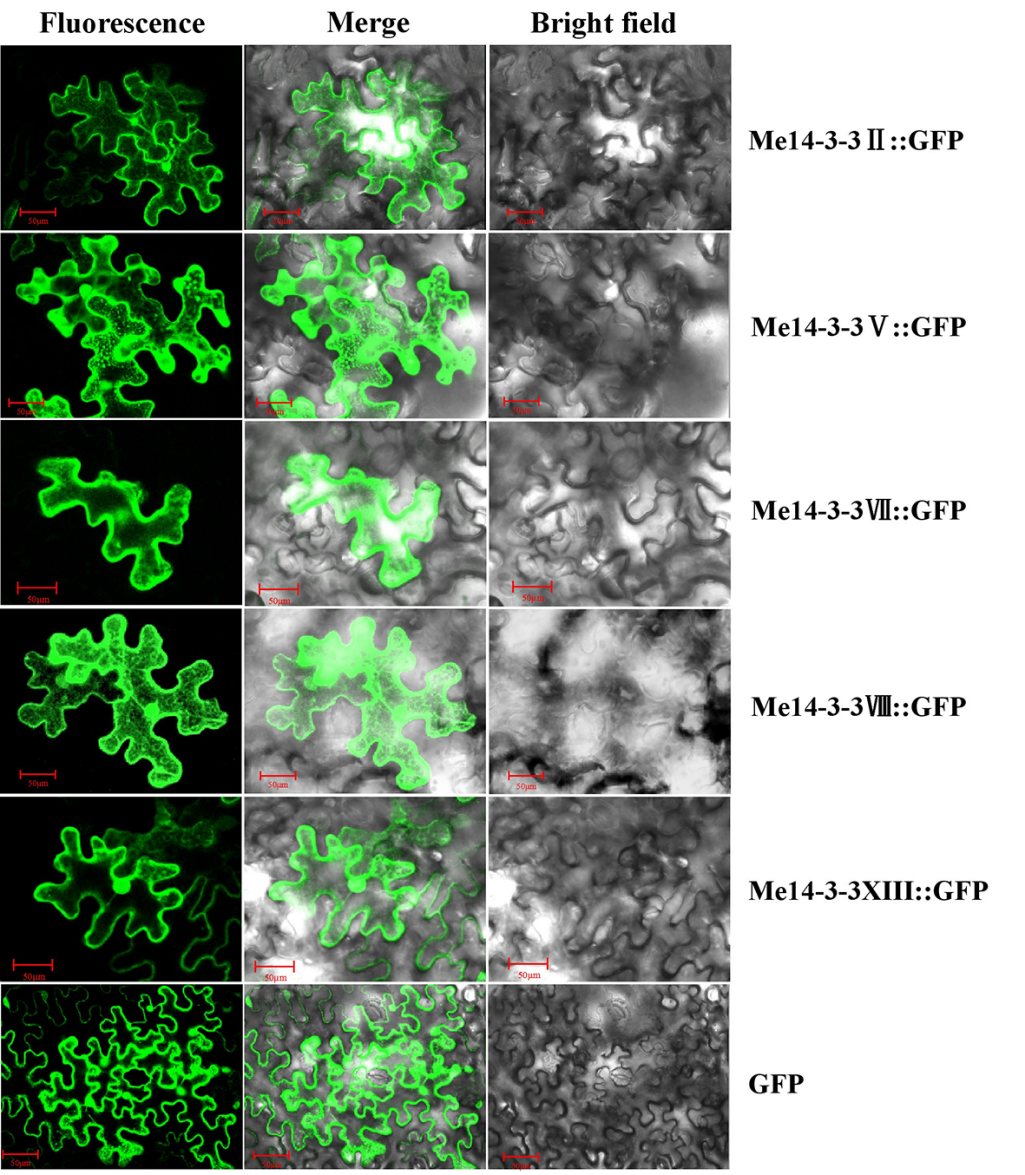
Subcellular locations of the Me14-3-3s-GFP fused proteins in epidermal cells of tobacco leaves. Green fluorescent protein (GFP) fluorescence was observed using the green channel (488 nm). Bar = 50 μm.

*Me14-3-3II*, *V*, *VII*, *VIII*, and *XIII* were the most highly expressed genes across all storage root development stages. Further, exploring the subcellular localization of these Me14-3-3 proteins may be helpful to understand their functions. Therefore, the CDS sequences of these genes were fused to the GFP gene and expressed downstream of the 35S promoter. These constructs were transiently expressed in tobacco leaves and observed after 72 h. Subcellular localization results showed that Me14-3-3II, V, VII, VIII, and XIII proteins were localized in the nucleus, cytoplasm, and plasma membrane (**Figure 4**).

### Functional assessment of *Me14-3-3II* to starch accumulation in the leaves of *A. thaliana*

**Figure 5:**
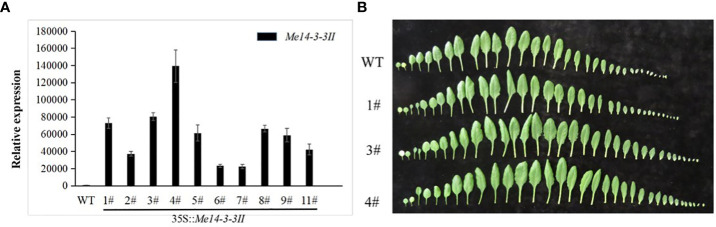
Phenotype and qRT-PCR validation of *Arabidopsis*. Me14-3-3II-overexpressing lines. **(A)** Expression levels of Me14-3-3II in wild-type plants and overexpressing lines. MeACTIN was the reference gene. Data are the mean ± SD of three independent assays. **(B)** Leaf number per plant in the WT and transgenic lines (1#, 3#, and 4#). Bar = 1 cm. WT, wild type.

**Figure 6:**
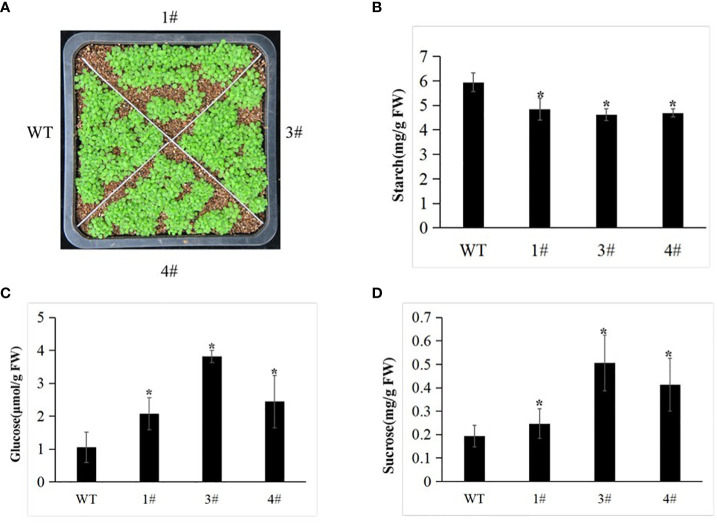
The WT and transgenic plants display different abilities. **(A)** Phenotype of the WT and selected transgenic lines (1#, 3#, and 4#) at the seedling stage. **(B)** Starch content of the WT and transgenic lines. **(C)** Sucrose content of the WT and transgenic lines. **(D)** Glucose content of the WT and transgenic lines. Data are the mean ± SD of three independent assays. * means the significant differences were determined by one-way ANOVA followed by Duncan’s multiple range test at p < 0.05. WT, wild type.

To assess the functions of *Me14-3-3II* responding to starch accumulation, overexpression of *Me14-3-3II* was transformed in *A. thaliana* ecotype Columbia (Col) *via* the floral dip method (**Figure 5**). The result showed that the contents of glucose and sucrose in the leaves of transgenic plants increased compared to those in wild-type (WT) plants (**Figure 6**). On the contrary, the starch content decreased in the leaves of transgenic plants (**Figure 6**), suggesting that *Me14-3-3II* is involved in the negative regulation of starch accumulation in *A. thaliana*.

### Functional assessment of *Me14-3-3II* to starch accumulation in cassava leaves and storage roots

**Figure 7:**
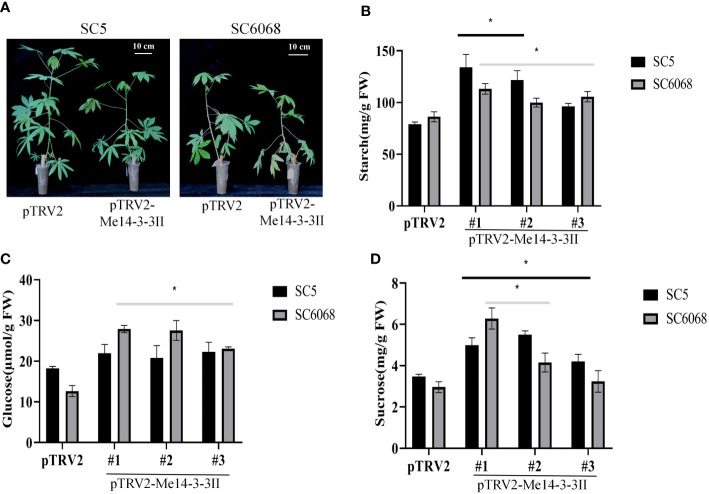
*Me14-3-3II* gene is involved in the negative regulation of starch accumulation in cassava leaves. **(A)** Phenotypes of cassava SC5 and SC6068 plants performed with VIGS of *Me14-3-3II*; pTRV2 was used as control. Bar = 10 cm. **(B)** Starch contents of leaves collected from cassava SC5 and SC6068 *Me14-3-3II*-silenced and control. **(C)** Sucrose contents of leaves collected from cassava SC5 and SC6068 *Me14-3-3II*-silenced and control. **(D)** Glucose contents of leaves collected from cassava SC5 and SC6068 *Me14-3-3II*-silenced and control. Data are the mean ± SD of three independent assays. * means the significant differences were determined by one-way ANOVA followed by Duncan’s multiple range test at p < 0.05. VIGS, virus-induced gene silencing.

To further verify the role of *Me14-3-3II* gene in starch accumulation in cassava, the expression of *Me14-3-3II* gene was inhibited by VIGS in cassava cultivars SC5 and SC6068 (**Supplementary Figure S1**; **Figure 7A**). The result showed that the contents of starch in *Me14-3-3II*-silenced (pTRV2-*Me14-3-3II*) SC5 and SC6068 leaves significantly increased compared to those of control (pTRV2) (**Figure 7B**). The contents of glucose and sucrose in both *Me14-3-3II*-silenced SC5 and SC6068 leaves also significantly increased with the exception of the sucrose content in *Me14-3-3II*-silenced SC5 #3 leaves compared to those in control (**Figures 7C, D**).

**Figure 8:**
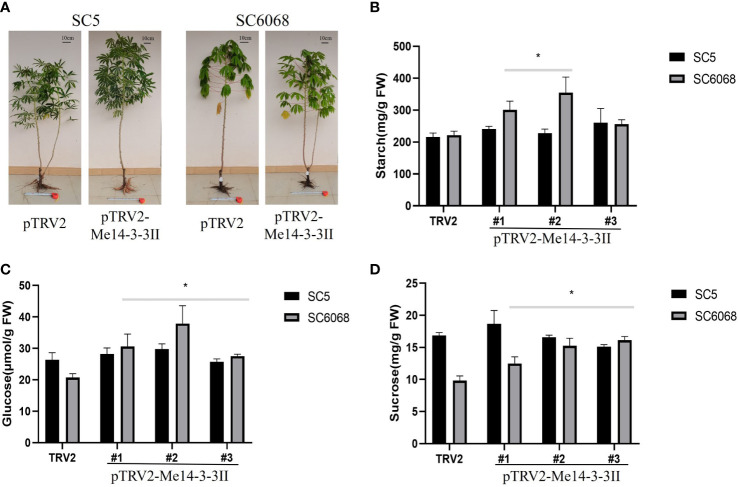
*Me14-3-3II* gene is involved in the negative regulation of starch accumulation in cassava storage roots. **(A)** Phenotypes of cassava SC5 and SC6068 plants performed with VIGS of *Me14-3-3II*; pTRV2 was used as control. Bar = 10 cm. **(B)** Starch contents of storage roots collected from cassava SC5 and SC6068 *Me14-3-3II*-silenced and control. **(C)** Sucrose contents of storage roots collected from cassava SC5 and SC6068 *Me14-3-3II*-silenced and control. **(D)** Glucose contents of storage roots collected from cassava SC5 and SC6068 *Me14-3-3II*-silenced and control. Data are the mean ± SD of three independent assays. * means the significant differences were determined by one-way ANOVA followed by Duncan’s multiple range test at p < 0.05. VIGS, virus-induced gene silencing.

To test the effects of *Me14-3-3II* gene on cassava storage roots, cassava SC5 and SC6068 *Me14-3-3II*-silenced seedlings were cultivated in the field and harvested after 4.5 months in the field (**Figure 8A**). The results showed that the starch contents in *Me14-3-3II*-silenced (pTRV2-*Me14-3-3II*) SC5 and SC6068 storage roots significantly increased compared to those of control (pTRV2) (**Figure 8B**). The contents of glucose and sucrose in *Me14-3-3II*-silenced SC5 were not significantly different compared to those in control; however, the contents of glucose and sucrose in *Me14-3-3II*-silenced SC6068 significantly increased compared to those in control (**Figures 8C, D**). The *Me14-3-3II*-silenced results from VIGS in both cassava leaves and storage roots further indicated that *Me14-3-3II* is involved in the negative regulation of starch accumulation.

## Discussion

The 14-3-3 protein plays a regulatory role in plant tricarboxylic acid cycle, glucose metabolism, shikimic acid synthesis, starch metabolism, root growth, and other physiological pathways by interacting with target proteins (Swatek et al., 2011; Gao et al., 2014; Zhang et al., 2019; Gao et al., 2021). When the repression of six *14-3-3* genes from potatoes was downregulated, nitrate and carbon fixation key enzymes from transgenic potato plants were activated and accelerated starch accumulation in the storage roots of transgenic plants (Żuk et al., 2003; Yu et al., 2016). Overexpression of a cassava *14-3-3* gene in *A. thaliana* confirmed that the older leaves of these transgenic plants contained higher sugar and starch contents than the wild-type leaves, suggesting that 14-3-3 proteins and their binding enzymes may play important roles in carbohydrate metabolism and starch accumulation during cassava root tuberization (Wang et al., 2016). A previous study showed that 15 different *14-3-3* genes (designated MeGRF1-15) were identified within the cassava genome. Based on evolutionary conservation and structural analyses, these cassava 14-3-3 proteins were grouped into the ϵ and non-ϵ clusters (Chang et al., 2020). In the present study, the prediction of the structure and function of cassava 14-3-3 proteins by bioinformatics shows that 16 cassava 14-3-3 proteins are structurally unstable and evolutionarily highly conserved hydrophilic protein families, in which the secondary structure of Me14-3-3 protein isomers is very similar. Compared with both sequences between MeGRF1-15 from Chang et al. (2020) and Me14-3-3I-XVI in the present study, we found 13 sequences that showed 100% similarity, while Me14-3-3IV and MeGRF15 displayed 90% similarity, and Me14-3-3XIV and MeGRF8 showed 99% similarity. However, Me14-3-3II did not have any similarity with the MeGRF family (**Supplementary Table S3**). Overexpression of *Me14-3-3II* in *A. thaliana* plants and VIGS of *Me14-3-3II* in cassava SC5 and SC6068 leaves and storage roots showed that *Me14-3-3II* was involved in the negative regulation of starch accumulation (**Figures 6** **–** **8**) in the present study. This result was opposite to that of Wang et al. (2016), indicating that 14-3-3 proteins have multiple functions, including playing important roles in cell signal transduction in response to starch accumulation.

As the main forms of carbohydrates, starch, glucose, and sucrose play a vital role in the balance and coordination of various carbohydrates (Li et al., 2022). Overexpression of *Me14-3-3II* in *A. thaliana* plants showed that leaf starch content decreased, but glucose and sucrose content increased. The VIGS of Me14-3-3II in SC5 and SC6068 indicated that along with the increased leaf starch content, so did the glucose in SC6068 and sucrose in SC6068 and SC5. However, when the starch content in SC6068 storage roots increased, the glucose and sucrose levels also rose, but the starch, glucose, and sucrose contents in SC5 storage roots did not have any changes. Starch is synthesized from ADP-glucose (ADPG), and in leaves, starch is synthesized in the chloroplasts, primarily in mesophyll cells. ADPG is made primarily in the cytosol, probably by sucrose synthase. However, there is disagreement about the subcellular compartmentation and pathway of ADPG synthesis. Fünfgeld et al. (2022) indicated that sucrose synthases are not involved in starch synthesis in *Arabidopsis* leaves. These results seemed to explain the reason that starch growth was not synchronized with that of glucose and sucrose in the present study.

By analyzing the subcellular localization process, it is inferred that Me14-3-3 protein is mainly distributed in the nucleus and cytoplasm, which is closely related to the function of 14-3-3 protein. From the results of phylogenetic tree analysis, there are two kinds of *14-3-3* genes in the ancestral genes of cassava. Most Me14-3-3 proteins have the typical nine domains; these protein structures are also found in other plants, such as in *Medicago truncatula* (Cheng et al., 2016), *H. brasiliensis* (Yang et al., 2014), tobacco (Chen et al., 2018), mulberry tree (Yan et al., 2015), and *Populus* (Tian et al., 2015). The tissue-specific expression patterns of *Me14-3-3* could provide a basis for understanding their functions in cassava plant development. Our results showed that *Me14-3-3* genes are widely expressed in various organs and tissues, such as leaves, midveins, lateral buds, OESs, FECs, FRs, SRs, RAMs, and SAMs. This suggests that these Me14-3-3 proteins in different organs and tissues may play a key role in cassava plants. The members of groups ϵ, *Me14-3-3IV*, and *Me14-3-3XI* with a close relationship have low expression levels in all tested samples. Two *Mt14-3-3* gene members of *Mt14-3-3b* and *Mt14-3-3j* from *M. truncatula* were specifically induced by salicylic acid (SA) and jasmonic acid (JA) (Cheng et al., 2016). The highly expressed *Me14-3-3* genes in tuber roots are *Me14-3-3IV* and *Me14-3-3XI*. It has been reported that 14-3-3 proteins from *Arabidopsis* regulated starch synthesis by the target starch synthase III family. Starch production in *Arabidopsis* leaves is limited through the inactivation of SSs by phosphorylation and 14-3-3 protein binding (Tetlow and Emes, 2015). The different-starch-content cassava cultivars (SC6068 and SC5) were used to explore the expression patterns of *Me14-3-3s* during tuber root development. Dramatically, *Me14-3-3* genes were highly expressed in a low-starch-content cultivar (SC6068) compared to SC5 during RIS, RES, and RMS. Many sucrose metabolism and starch synthesis-related enzymes are involved in cassava tuber root development, such as invertase, sucrose synthase, starch synthase, and starch-branching enzyme (Swatek et al., 2011). It has been reported that 14-3-3 proteins negatively regulated the activity of these enzymes in other plants (Gao et al., 2014; Tetlow and Emes, 2015). Sucrose synthase activity was shown to be inhibited by exogenous 14-3-3 in a dosage-dependent manner (Morris, 2010). Proteins interacting specifically with 14-3-3 proteins were analyzed by sodium dodecyl sulfate–polyacrylamide gel electrophoresis (SDS-PAGE) and Western blotting. These assays showed that starch synthase I (SSI), starch synthase II (SSII), starch-branching enzyme IIa (SBEIIa), starch-branching enzyme IIb (SBEIIb), and ADP glucose pyrophosphorylase large subunit (SH2) interacted with 14-3-3 proteins (Song et al., 2009). Reduction of the subgroup of *Arabidopsis* 14-3-3 proteins by antisense technology resulted in a two- to fourfold increase in leaf starch accumulation (Sehnke et al., 2001). Therefore, we can speculate that 14-3-3 proteins may play a key role in regulating starch accumulation in cassava tuber roots. Ten 14-3-3 proteins from *H. brasiliensis* were targeted exclusively to the nucleus and cytoplasm (Yang et al., 2014). Similarly, Me14-3-3II, V, VII, VIII, and XIII proteins, which were highly expressed across all tuber root development stages, were localized to the nucleus and cytoplasm (**Figure 6**). How the Me14-3-3 proteins regulate starch accumulation during storage root development will be further investigated.

## Conclusion

In this study, a genome-wide analysis of the *14-3-3* gene family was characterized with a particular focus on Me14-3-3II response to starch accumulation in cassava storage roots. A total of 16 *Me14-3-3* genes were identified and characterized. Phylogenetic relationship, gene structures, subcellular localization, gene expression in various tissues, and different cassava cultivars of 14-3-3s were analyzed. Moreover, overexpression of *Me14-3-3II* in *A. thaliana* and VIGS in cassava analyses unveiled that *Me14-3-3II* genes may play a crucial role in the negative regulation of starch accumulation in cassava leaves and storage roots. Overall, the results provide new information for the improvement of cassava cultivars in increasing starch content through molecular breeding.

## Data availability statement

The original contributions presented in the study are included in the article/**Supplementary Material**. Further inquiries can be directed to the corresponding author.

## Author contributions

RP, YW, and FA: conceptualization and original draft preparation. YW, AF, and YY: software, review and editing, and visualization. JX: methodology and project administration. SC and XL: validation form analysis. XL: data curation. WZ: investigation and resources. SC and HL: modification and supervision. SC: funding acquisition. All authors have contributed to the article and approved the submitted version.

## Supplementary material

Supplementary Table 1 The gene names and locus IDs of these 14-3-3 proteins.

Supplementary Table 2 Fragments per kilobase of transcript per million mapped reads (FPKM) values of the *Me14-3-3* genes in 11 tissues of cassava cultivar TME204. The first worksheet was the FPKM values; the red color represented expression levels >150 folds; the yellow color showed that the expression levels were equal to 50–150 folds; and the green color meant expression levels <50 folds. The second worksheet was the log_2_FPFM values.

Supplementary Table 3 The accession numbers of 14-3-3 proteins from cassava (*M. esculenta*), Arabidopsis (*Arabidopsis thaliana*), poplar (*Populus trichocarpa*), rice (*Oryza sativa*), alfalfa (*Medicago truncatula*), soybean (*Glycine max*), grass (*Brachypodium distachyon*), sorghum (*Sorghum bicolor*), and rubber (*Hevea brasiliensis*).

Supplementary Table 4 Specific primer sequence of the *Me14-3-3* genes.

Supplementary Figure 1 Expression levels of Me14-3-3II in wild-type plants and cassava SC5 and SC6068 VIGS lines. Data are the mean ± SD of three independent assays.
