# Supplementary figures and images for: Genome-wide identification and characterization of 14-3-3 gene family related to negative regulation of starch accumulation in storage root of Manihot esculenta
Source: Front Plant Sci. 2023 Aug 29;14:1184903. doi: 10.3389/fpls.2023.1184903 (PMC10497974; doi:10.3389/fpls.2023.1184903)

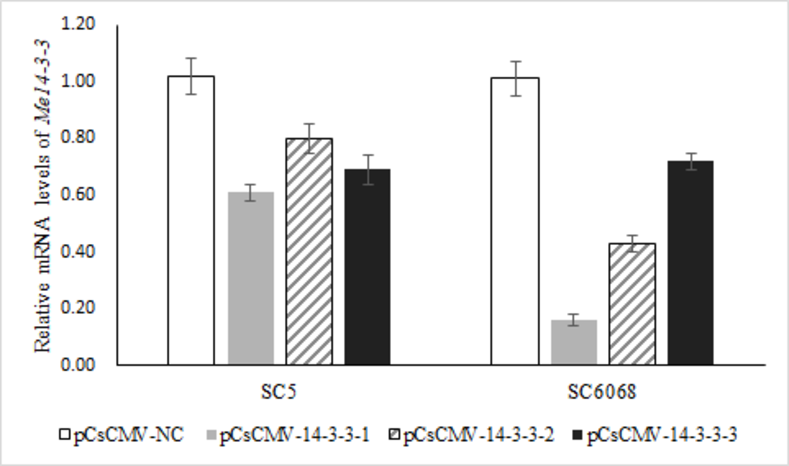

Supplement: Supplementary Figure 1 — Expression levels of Me14-3-3II in wild-type plants and cassava SC5 and SC6068 VIGS lines. Data are the mean ± SD of three independent assays. [file Image_1.tif]
